# Association Between Socio-Political and Economic Factors and COVID-19 Vaccination Uptake: US–Mexico Border Study

**DOI:** 10.3390/epidemiologia7020045

**Published:** 2026-04-01

**Authors:** Komla Koumi, Soyoung Jeon, Yu-Feng Lee

**Affiliations:** 1 Center for Business and Economic Research, Culverhouse College of Business, The University of Alabama, Tuscaloosa, AL 35401, USA; 2 Department of Economics, Applied Statistics and International Business, New Mexico State University, Las Cruces, NM 88003, USA; sjeon@nmsu.edu (S.J.); wlin@nmsu.edu (Y.-F.L.)

**Keywords:** COVID-19 vaccination, U.S.–Mexico border counties, socioeconomic determinants, political affiliation, race and ethnicity

## Abstract

Background/Objectives: The implementation of COVID-19 vaccination in the United States has revealed substantial disparities driven by geography, socioeconomic conditions, and political ideology. This study examines the association between these factors and COVID-19 vaccination uptake across 360 counties in four U.S.–Mexico border states, characterized by distinct socio-political traits. Methods: Using county-level data, this study employed multivariable regression analysis and GIS mapping to assess the effects of income, education, employment, age, race, ethnicity, occupation, metropolitan status, border status, and political affiliation on Dose 1, Dose 2, and booster vaccination rates. Results: The analysis showed that Dose 1 vaccination rates were significantly higher in border counties and metropolitan areas. Democratic population share and per capita income were positively associated with vaccination uptake. Dose 2 vaccination rates exhibited patterns similar to those observed for Dose 1. Booster vaccination rates were positively associated with Democratic affiliation, the proportion of the population with at least a high school education, and the share of individuals aged 65 years and older. In contrast, unemployment rates were negatively associated with booster uptake. Racial and ethnic composition was also associated with vaccination outcomes: higher Black population shares were associated with lower Dose 1 vaccination rates, whereas higher Native American population shares were associated with higher vaccination rates. Booster uptake was higher with larger shares of the Asian population but slightly lower with larger shares of the White population. Conclusions: COVID-19 vaccination uptake in U.S.–Mexico border counties was associated with a complex interaction of geographic, socioeconomic, demographic, and political factors. These findings underscore the importance of targeted, context-specific public health strategies to reduce vaccination disparities and improve booster coverage in border regions.

## 1. Introduction

The COVID-19 pandemic revealed substantial geographic and demographic disparities in vaccination uptake across the United States, driven by socioeconomic, political, and structural factors [1,2,3,4]. These disparities are particularly evident in the U.S.–Mexico border region, a geographically and socio-politically distinct area marked by high cross-border mobility, economic interdependence, immigration-related vulnerabilities, and historical healthcare disparities [5,6,7,8,9]. As such, the border region provides a critical setting for examining how place-based structural conditions shape vaccination behavior.

The border states, comprising Arizona, California, New Mexico, and Texas experienced noticeable variation in COVID-19 vaccination coverage. As of April 2023, full vaccination rates ranged from over 75% in New Mexico and California to just over 64% in Texas [10]. Such disparities were even more pronounced within the border counties where limited healthcare access, lower economic opportunity, and heightened exposure risks intersected, such as in Texas, where border-county vaccination rates fell to 50%, indicating deep intra-state inequities [11]. Moreover, border governance measures such as travel restrictions and port closures, while intended to slow down the spread of COVID-19, created socio-economic disruptions that further complicated public health interventions [12].

Recent studies have highlighted the various aspects of socio-political conditions, including immigration status, historical distrust in the healthcare system, political ideologies, and economic disparities, which influenced vaccination outcomes in border communities [13,14]. However, despite the growing relevant literature, there remains a high need for more targeted and data-driven research on the U.S.–Mexico border. To address this gap, this study examines county-level socioeconomic, demographic, political, and geographic factors associated with COVID-19 vaccination across 360 counties in the four U.S.–Mexico border states. It extends prior research [4] by analyzing vaccination outcomes, including the first-dose, second-dose, and booster-dose vaccinations as of 14 September 2022. Using regression analysis and GIS-based spatial assessment, the study investigates how factors such as income, employment, education, race, age structure, occupation, political affiliation, metropolitan status, and border location are associated with vaccination uptake across different stages of the vaccination campaign. By focusing on the U.S.–Mexico border as a unique and policy-relevant setting, this study contributes to a deeper understanding of how structural and contextual factors influence vaccination behavior over time, providing insights for targeted public health strategies in border and similarly complex regions.

### 1.1. The COVID-19 Pandemic and the Border Paradox

The COVID-19 pandemic has reignited critical discussions about the role of borders in public health governance, particularly in regions characterized by high mobility and cross-border dependence. In the U.S.–Mexico border region, containment measures such as border closures and mobility restrictions produced uneven public health outcomes, disproportionately impacting marginalized populations and cross-border communities, thereby deepening existing healthcare disparities [12]. Evidence suggests that U.S. border counties experienced higher standardized mortality ratios than the national average, while mortality rates across U.S. border states were negatively correlated with the Human Development Index (HDI), underscoring the intersection of health vulnerability and socio-economic disadvantage [14]. County-level analyses in Texas further revealed higher COVID-19 fatality rates in border counties relative to interior counties, highlighting persistent spatial inequities in health outcomes [15].

Recent studies focused on the unique vulnerabilities faced by border populations during the pandemic. A community-based COVID-19 testing program in a predominantly Latino neighborhood near the U.S.–Mexico border points out the importance of culturally tailored health interventions to address testing inequities [16]. Another study on cross-border truck drivers revealed the logistical and communicative challenges in reaching mobile populations with prevention messaging [17]. In response to healthcare disruptions, researchers proposed post-pandemic strategies to improve healthcare access for U.S.–Mexican migrants using the Mexican health system [6]. Furthermore, analysis of vaccine uptake in a border community revealed sociocultural and economic factors influencing vaccination rates, shedding light on persistent disparities in public health engagement [7].

Further studies have shown that the COVID-19 pandemic deepened health inequities across the U.S.–Mexico border region, disproportionately affecting Hispanic and Latino communities. Among Mexican and Central American immigrants, factors such as undocumented status, lack of health insurance, and fear of engaging with healthcare systems significantly hindered access to essential services during the pandemic [18]. In El Paso County at the Texas–Mexico border, longstanding constraints related to healthcare access and socioeconomic vulnerability further intensified these challenges [19]. Additionally, restrictive border policies and reduced cross-border mobility further complicated access to health services and vital resources for transborder populations, revealing the critical intersection between public health and mobility in borderland contexts [13].

### 1.2. Public Perception and Vaccine Acceptance in Border Contexts

Public perception of COVID-19 and trust in vaccination have significantly influenced the course of the pandemic response. The willingness to get vaccinated tends to increase among communities with stronger trust in government and science, reduced safety concerns, lower costs, and access to reliable vaccine-related information [20,21,22,23]. Conversely, misinformation about vaccine safety and the speed of vaccine development has fueled hesitancy in various populations, especially those with historical skepticism toward public health authorities [24,25]. Throughout the United States, perceptions of the virus’s severity and trust in health institutions have varied across demographic, geographic, and political lines, influencing vaccine acceptance and uptake [26,27]. Trust in local versus federal authorities also matters. In border regions, where policy enforcement and communication often reflect state and federal dynamics, inconsistencies can weaken public confidence [28]. Research highlights that misinformation and longstanding distrust of healthcare institutions disproportionately affect marginalized border populations, reinforcing disparities in vaccine acceptance and coverage [29,30,31]. Additional research across various vaccine platforms has underlined unique advantages and limitations [32,33,34], while broader studies emphasize the health, economic, and social benefits of vaccination, supporting widespread immunization [35,36].

### 1.3. Economic and Socio-Political Impacts on Vaccination Efforts

Socio-economic and political conditions have been shown to significantly influence COVID-19 vaccination patterns. Racial and ethnic minority communities, particularly Hispanic populations concentrated in border counties, faced compounded barriers to vaccination, including limited healthcare access, employment constraints, and transportation challenges [4,37,38]. At the county level, variations in political ideology have influenced public health messaging and vaccine uptake. Studies have found that counties with higher proportions of conservative voters have lower vaccination rates, reflecting skepticism toward federal public health initiatives and resistance to vaccine mandates [1,2,3,4,39,40]. This political schism has resulted in inconsistent implementation of public health strategies and uneven risk communication, which hampers efforts to increase vaccine coverage, particularly in communities with strong political polarization. Furthermore, education played a crucial role; individuals with only a secondary education or those who had discontinued their academic studies exhibited greater vaccine hesitancy, whereas those with higher levels of education were more likely to get vaccinated.

### 1.4. Theoretical Linkage

**Figure 1:**
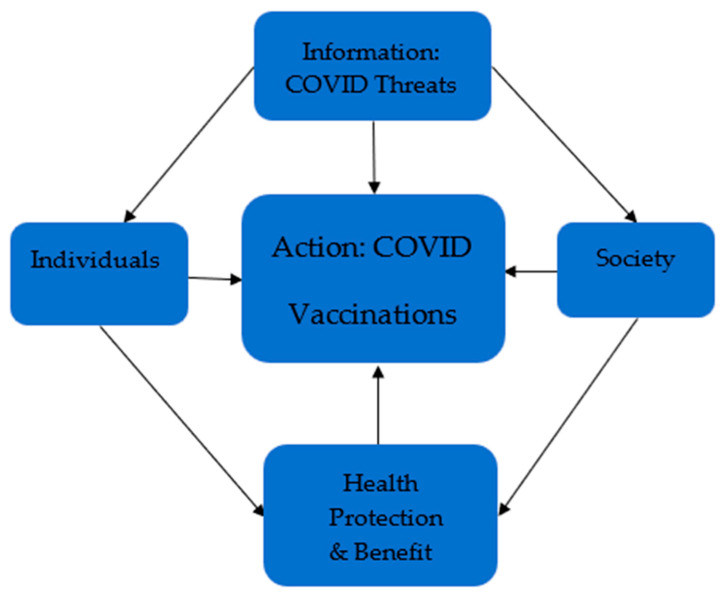
Rational Choice Theory (RCT) Application to COVID Vaccination and Impact. Source: Authors’ design.

This study draws on the Theory of Border (ToB) and Rational Choice Theory (RCT) to guide variable selection and interpret vaccination patterns along the U.S.–Mexico border. Proposed by Thomas Nail, ToB conceptualizes the border as a dynamic geographic and socio-political space characterized by mobility, interdependence, and hybridity [41]. The U.S.–Mexico border uniquely generates interdependence through economic, social, and cultural interactions, creating structural and community dynamics that shape health behaviors during the COVID-19 pandemic. In the models, these dynamics are captured through border county status, reflecting the influence of structural conditions, cross-border mobility, and community networks on vaccination uptake. The movement-oriented ‘kinopolitics’ and bi-national workforce hybridity further highlight how diverse social and cultural groups may respond differently to public health interventions. Complementing this, RCT, originally proposed by Adam Smith, frames individuals as rational agents who weigh costs and benefits when making health decisions [42]. As shown in Figure 1, in the presence of a pandemic, the U.S.–Mexico border potentially exposes inhabitants to higher risks of cross-border virus spread, prompting them to choose and take vaccination for their individual and communal health protection. This framework motivates the inclusion of access- and utility-related variables that shape perceived costs and benefits, such as metropolitan status, socioeconomic indicators, and age structure, in the analysis. It guides the interpretation of differences between primary-series and booster uptake as a rational response to elevated risk and collective benefit.

## 2. Materials and Methods

### Data Sources and Variables

This study extends our previously published analysis [4]. The data sources, study population, covariate definitions, and regression modeling framework are identical to those described in detail in the prior work and are therefore summarized only briefly here.

The present analysis introduces two key extensions: (1) the inclusion of border county status and metropolitan status as additional geographic exposures and (2) the estimation of separate models for three COVID-19 vaccination outcomes: receipt of at least one dose, completion of a primary vaccination series, and receipt of a booster dose. All models follow the same specification and estimation procedures as in the prior study.

County-level COVID-19 vaccination rates, containing percentage of individuals who were vaccinated with at least one dose (Dose 1), completed a primary series (have second dose of a two-dose vaccine or one dose of a single-dose vaccine; Dose 2), and completed a primary series and received a booster (Booster) as of 14 September 2022, were analyzed in this study. This cutoff date was chosen to capture approximately one year of booster uptake following the initial booster recommendation for adults 65 years and older issued in September 2021 [43]. The characteristics of each vaccine type are summarized in Table A1 of the Appendix A.

The analyses draw on multiple data sources and are based on an aggregated dataset of 360 counties across the four-border states (AZ, CA, NM, and TX) in the Mid- and Southwest regions. Due to missing vaccination data, 352 counties are included in the sample, covering the period from the onset of the COVID-19 pandemic through mid-2022. County-level socio-economic and political covariates and their respective data are described below and summarized with sources in Table 1.

## 3. Variable Description

Variables in this study include state, age structure, occupation, education, income, race/ethnicity, and political composition, with particular emphasis on border status and metropolitan status as key geographic determinants of vaccination coverage.

Border Area: Border counties are defined according to the La Paz Agreement as counties located within 100 km of the U.S.–Mexico border. A total of 44 counties are classified as border counties, while the remaining 316 are classified as non-border counties. Eight non-border counties in California were excluded due to missing data, resulting in a final sample of 352 counties [45].

Metro Area: Metropolitan areas follow the U.S. Office of Management and Budget definition of areas as counties with over 50,000 residents with economic integration. The CDC classifies counties as metropolitan or non-metropolitan and reports vaccination rates accordingly. Prior studies show that non-metro areas have lower vaccine access and uptake [52,53]. Based on this result, we assume that the non-metropolitan countries would have less access to the vaccine.

### Statistical Methods

**Figure 2:**
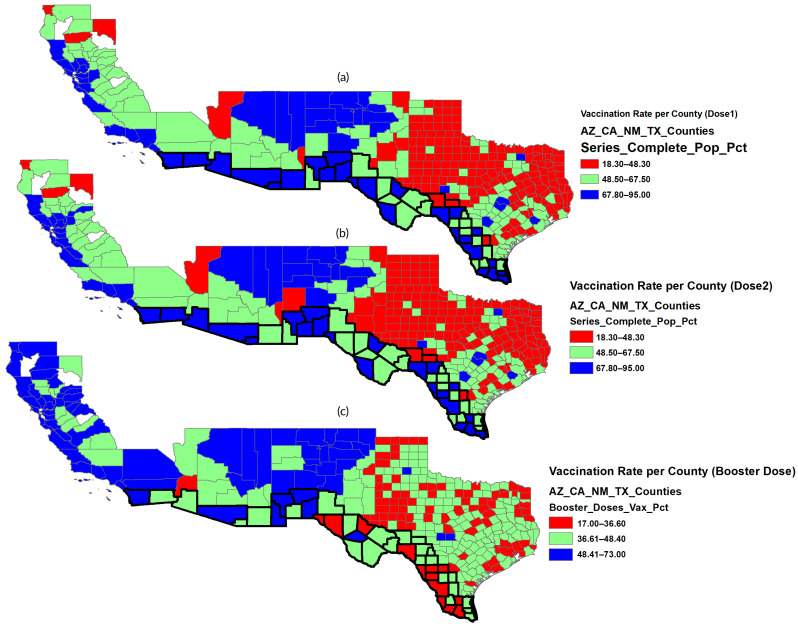
Geographic distribution of COVID-19 vaccination rate as of 14 September 2022; (**a**) Dose 1, (**b**) Dose 2, and (**c**) Booster. Border Counties are outlined in black. Source: Authors’ design using ArcGIS (Version 10.8).

**Figure 3:**
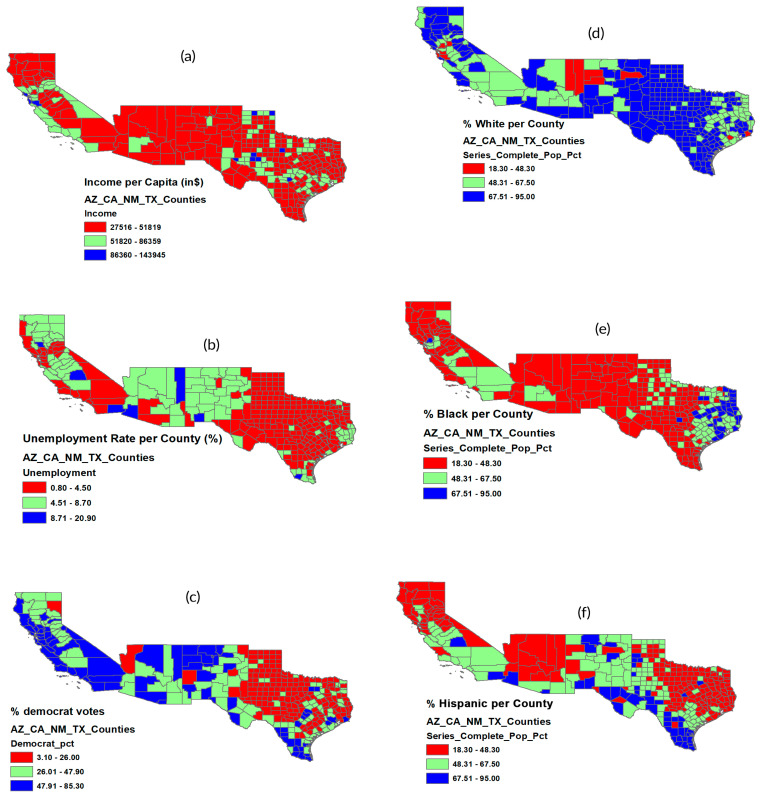
Geographic distribution COVID-19 vaccination rate (Dose1, Dose2 and Booster doses as of 14 September 2022, economic status described by (**a**) income per capita, (**b**) unemployment rate, (**c**) political affiliation, and racial and ethnicity effects described by (**d**) percent of White population, (**e**) percent of Black population, and (**f**) percent of Hispanic population. Source: Authors’ design using ArcGIS (Version 10.8).

ArcGIS (version 10.8) was employed to visualize the spatial distribution of vaccination rates and to examine how socioeconomic and political variables influenced COVID-19 vaccination patterns across counties and states (see Figure 2 and Figure 3). The spatial distribution below (Figure 2) shows that Dose 1 and Dose 2 vaccination rates are generally higher in border counties than in non-border counties. Booster uptake, however, shows less consistent differences between the two groups, with some border counties having lower rates than their non-border counterparts.

Figure 3 illustrates how socioeconomic and demographic factors influence vaccination uptake across U.S. counties, as analyzed in Figure 2. Higher rates of Dose 1 and Dose 2 are associated with greater per capita income and stronger Democratic affiliation. Booster uptake follows a similar pattern, showing a positive association with Democratic-leaning counties and a negative association with higher unemployment. Racial and ethnic patterns reveal that counties with a larger Black population tend to have lower Dose 1 coverage. At the same time, those with a higher share of Hispanic residents show increased vaccination uptake. Booster coverage slightly decreases in counties with a higher percentage of White residents.

We estimated linear regression models for county-level vaccination rates (Dose 1, Dose 2, and Booster). A baseline model (Model 1, M1) included nine socioeconomic and political covariates retained after multicollinearity assessment. The percentage of the population aged 65 years and older was added in Model 2 (M2), and racial/ethnic population shares (percentage of White/Black/Asian/Native American/Hispanic population) were included separately in Model 3 (M3.1–M3.5). Three types of vaccination rates (i = Dose 1, Dose 2, Booster) are described by the following models:

**M1.**  *vaccination rate_i_ = *$\beta_{0}$*+ *$\beta_{1}$*state + *$\beta_{2}$*metro status + *$\beta_{3}$*border county + *$\beta_{4}$*unemployment + *$\beta_{5}$*democrat_pct + *$\beta_{6}$*farmworker + *$\beta_{7}$*rural_pct + *$\beta_{8}$*HS graduate + *$\beta_{9}$*income*,

**M2.**  *vaccination rate_i_ = *$\beta_{0}$*+ *$\beta_{1}$*state + *$\beta_{2}$*metro status + *$\beta_{3}$*border county + *$\beta_{4}$*unemployment + *$\beta_{5}$*democrat_pct + *$\beta_{6}$*farmworker + *$\beta_{7}$*rural_pct + *$\beta_{8}$*HS graduate + *$\beta_{9}$*income + *$\beta_{10}$*age_65over*,

**M3.**  *vaccination rate_i_ = *$\beta_{0}$*+ *$\beta_{1}$*state + *$\beta_{2}$*metro status + *$\beta_{3}$*border county + *$\beta_{4}$*unemployment + *$\beta_{5}$*democrat_pct + *$\beta_{6}$*farmworker + *$\beta_{7}$*rural_pct + *$\beta_{8}$*HS graduate + *$\beta_{9}$*income + *$\beta_{10}$*age_65over* + $\beta_{11}$*race/ethnicity.*

**Figure 4:**
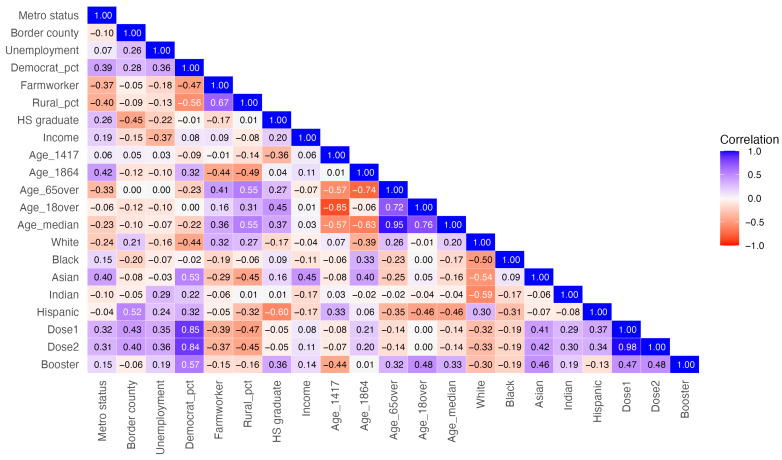
Pairwise correlation among socioeconomic variables.

Multicollinearity was assessed using pairwise correlations (Figure 4), variance inflation factors (VIFs), and condition indices. Although some predictors exhibit moderate pairwise correlations, model-level diagnostics indicate no evidence of harmful multicollinearity: all VIFs were below 4, and the largest condition indices across models ranged from 3.44 to 3.97, below conventional thresholds of concern. All statistical analyses were conducted using Stata (version 16) and R (version 4.1.0). The level of significance was set at *p* < 0.05.

## 4. Results

The descriptive patterns of vaccination uptake and socioeconomic characteristics across the four border states are presented to establish the broader context for county-level differences. Table 2 summarizes the descriptive statistics for socioeconomic characteristics and vaccination rates for all 352 counties in four states and each county per state. As of 14 September 2022, the average percentage of fully vaccinated individuals was 52.61%, with Texas having the lowest (47.64%) and California the highest (65.87%) of the four border states. The rate of booster-dose vaccination was also the lowest (37.56%) in Texas and the highest (53.54%) in California.

California and Texas are two of the largest and most populous states in the United States, but they differ significantly in terms of demographics, political leanings, and industries. California has a higher percentage of racial minorities, including large Asian (9.10%) and Black (4.02%) communities, while Texas has a diverse population with a higher percentage of White (88.61%), Black (6.88%), and Hispanic (35.46%). California counties had the highest Democratic voter turnout (55.04%), while Texas counties had the lowest Democratic population (24.53%). California is more urbanized, with large metropolitan areas (74% in CA vs. 32.3% in TX). Texas has a mix of urban and vast rural areas (rural percentage: 55.52% in TX vs. 20.57% in CA), with the highest percentage of farm workers (6.39%) among the four states.

New Mexico and Arizona had higher rates of at least one dose of vaccine (76.46% in NM, 76.31% in AZ). The unemployment rate was the highest in Arizona (6.64%), and both states had a higher percentage of population aged ≥ 65 years (22.15% in NM, 21.70% in AZ) and lower income per capita ($40,855 for NM, $40,592 for AZ) than CA and TX. Arizona and New Mexico have a significant presence of the Native American population (15.10% in AZ, 9.32% in NM).

To expand on the descriptive patterns, regression analysis is conducted to determine county-level factors influencing vaccination. The associations of county-level characteristics obtained from the regression models for the three types of vaccination rates (Dose 1, Dose 2, and Booster) are described in Table 3, Table 4 and Table 5, respectively. Continuous predictors were mean-centered, and effect sizes are reported as changes in vaccination rates associated with an interquartile range (IQR) increase to facilitate interpretability and comparison across variables. In Model 1 for Dose 1 vaccination, where age and race variables were not included, the county’s state, border status, percentage of Democrats, and income per capita were found to be significantly related to the Dose 1 vaccination rate of the county (R^2^ = 0.792; see M1 in Table 3). Dose 1 rates were higher in border counties than in non-border counties, and increases in vaccination rates were associated with increased Democrat vote percent and per capita income.

In addition to the significant factors found in M1, the metro status variable was also significant in a model (M2) that included the percentage of county population aged 65 and older (R^2^ = 0.793). The Dose 1 vaccination rates were higher in metro counties than in non-metro counties (=2.246, 95% confidence interval (CI) = 0.107–4.384) and also in border counties than in non-border counties (=11.184, 95% CI = 8.005–14.363). The vaccination rates increased by 17.45% (95% CI = 15.47–19.46) with a change from the 25th to the 75th percentile (i.e., IQR change) in the Democratic population and by 1.23% (95% CI = 0.44–2.01) for an IQR (=$12,925.25) increase in per capita income in the county.

The association of racial composition and rurality with vaccination rates is subsequently examined, assessing how demographic structure shapes outcomes beyond socioeconomic and political factors. The models M3.1–M3.5 in Table 3 show that the vaccine rates increased with decreasing rates of the Black population (=−0.386, 95% CI = −0.547–−0.226) and increasing percentages of Native Americans (=0.330, 95% CI = 0.195–0.465). Unlike the other models, the percentage of the rural population in M3.4 was significantly associated with vaccination rates. The White, Asian, and Hispanic population percentages were not significant in predicting the county’s Dose 1 vaccination rate.

Model 1 for vaccination rates of Dose 2 showed the same significance result as M1 for Dose 1 (see Table 4). The county’s state, border state, percentage of Democrats, and per capita income were significant factors in M1 (R^2^ = 0.760), while the metro variable was not significant in M2 (R^2^ = 0.760). In M2, the Dose 2 vaccination rate was higher in border counties than in non-border counties ($\beta_{3}$ = 8.936, 95% CI = 5.819–12.053). The vaccination rates increased by 16.56% (95% CI = 14.61–18.48) with an IQR change in the Democratic population and by 1.43% (95% CI = 0.66–2.20) with an IQR change in per capita income in the county. The race/ethnicity variable was significant in the Black population (M3.2) and Native American models (M3.4), with an IQR change in the Black population associated with a 2.23% (95% CI = 1.30–3.15)decrease in Dose 2 rates and an IQR change in the Native American population associated with a 0.39% (95% CI = 0.25–0.53) increase. In the model M3.4, metropolitan status was significant, with higher vaccination rates in metro counties than in non-metro counties (β_2_ = 2.412, 95% CI = 0.351–4.473).

The regression models for booster vaccination rates showed different associations than the Dose 1 or Dose 2 models. For the model that included Age ≥ 65 years old (M2, Table 5), a county’s state, unemployment rate, percentage of Democrats, percentage of high school graduates, and percentage of people 65 and older were significantly associated with receiving a booster vaccine (R^2^ = 0.744). The booster vaccination rate increased by 3.80% (95% CI = 2.87–4.74) per an IQR change in the elderly population in the county. The model shows that for every IQR change in the unemployment rate, the booster vaccine rate decreases by 0.66% (95% CI = 0.12–1.20), while an IQR change in the Democratic population and the high school graduate population increases the rate by 5.58% (95% CI = 4.42–6.75) and 1.55% (95% CI = 0.77–2.32), respectively.

White and Asian population percentages were significant in explaining the county’s booster vaccination rate in Model 3 (M3). The White model, M3.1 in Table 5, with R^2^ = 0.749 shows that the booster rate decreased by 0.74% (95% CI = 0.12–1.36) with an IQR change in the White population. On the other hand, model M3.3 shows that an IQR change in the Asian population is associated with a 0.43% (95% CI = 0.26–0.59) increase in booster vaccination rates. Another thing to note is that the unemployment rate was not significant in the Asian model. 

## 5. Discussion

The county’s State, the percentage of Democrats, and per capita income were all important factors that were consistently associated with vaccination rates, regardless of vaccination type (Dose 1 & Dose 2). This finding aligns with a previous research examining full vaccination rates in counties in five states (Arizona, Colorado, New Mexico, Oklahoma, and Texas) [4]. However, unlike the previous study, the current analysis found that the percentage of the elderly aged 65 and over did not present a significant impact on Dose 1 and Dose 2 vaccination rates, suggesting that age-related prioritization alone did not drive early vaccine uptake at the county level once border-related factors were accounted for.

This study notably found that border status was a significant factor contributing to both Dose 1 and Dose 2 vaccination rates, indicating higher vaccination rates in border counties than in non-border counties This pattern is consistent with evidence that early COVID-19 vaccination in border regions was facilitated by cross-border mobility requirements and targeted outreach that enforced vaccination compliance and improved access to vaccination services, thereby promoting uptake of the primary vaccine series [54,55]. In addition to structural access considerations, higher vaccination rates in border counties may reflect local public health initiatives, culturally tailored campaigns, and stronger community networks that mitigated geographic and logistical barriers. While Dose 1 vaccination rates were higher in metropolitan counties than in non-metropolitans, the Dose 2 rate did not differ significantly between them. Consequently, targeted strategies such as removing barriers to vaccine access may be implemented to increase the vaccination completion rate in non-border and nonmetropolitan counties. As healthcare access density was not included in the models, high vaccination in border counties may also reflect targeted state and federal outreach efforts rather than demographics alone; future analyses could incorporate these programs to better distinguish structural access from social and political factors. In contrast to full-series vaccine uptake, our findings show that booster vaccination followed a distinct pattern. In the booster model, the county’s unemployment rate, percentage of high school graduates, and percentage of the population aged 65 years or older were significant factors besides the percentage of Democrats. In contrast, per capita income was not associated with the rates of booster vaccination. This suggests that income-related access barriers, which were significant earlier in the vaccination campaign, played a more minor role once boosters became widely available, with uptake driven more by perceived risk, eligibility, and behavioral factors [56,57]. Metro or border status did not have a significant effect on booster vaccination rates, suggesting that geographic access was less influential in determining booster vaccination.

As summarized in Table A1, booster doses are administered after completion of the primary vaccine series to help maintain immunity over time and address emerging variants. Unlike the initial vaccine rollout, booster distribution began later in the pandemic, in fall 2021, when vaccine hesitancy, pandemic fatigue, and shifts in public health messaging had become more prominent. These contextual differences likely contributed to changes in the predictor’s significance. For example, the CDC’s September 2021 recommendation prioritized adults aged 65 and older for booster doses, with eligibility gradually expanding to younger age groups by November 2021 [43]. This policy explains the strong positive association between the percentage of older adults and booster vaccination rates observed in the regression model.

Regarding the effects of county-based racial composition on vaccination for Dose 1 and Dose 2, vaccination rates were positively associated with the proportion of Native Americans, but negatively associated with the proportion of the black population, while the rates stayed neutral around the Hispanic population. The higher vaccination rates in counties with larger Native American populations likely reflect the success of community-led and tribal health system initiatives, which employed culturally tailored outreach, pop-up clinics, and trusted messengers to increase vaccine access and acceptance [58,59]. In contrast, lower rates in counties with larger Black populations may reflect systemic barriers to access and historical mistrust in healthcare [60,61]. The neutral association with Hispanic populations reflects heterogeneity across border counties, where immigration status, language access, occupational risk, and cross-border mobility interact [62,63]. Furthermore, in the booster model, higher vaccination rates were observed in counties with larger percentages of the Asian population and smaller percentages of the White population. It is essential to consider the potential for selection bias when interpreting booster vaccination patterns, as individuals receiving booster doses constitute a selected subgroup—those who have already completed the primary vaccine series. This selection may amplify differences related to health-seeking behavior, trust in science, and sustained engagement with preventive care, rather than reflecting initial access constraints, thereby shaping observed racial patterns in booster uptake. This study employs an ecological design based on county-level aggregated data. Accordingly, the observed associations between sociodemographic characteristics and vaccination rates should not be interpreted as reflecting individual-level behavior or causal relationships. In particular, county-level measures of a population’s race/ethnicity, socioeconomic characteristics, and farmworker status may obscure substantial within-county heterogeneity and therefore are subject to ecological fallacy. For example, higher vaccination rates in counties with larger proportions of certain racial or ethnic groups do not imply that individuals within those groups were more likely to be vaccinated.

In addition, several covariates, including racial/ethnic composition, income, and farmworker population, are measured at different points in time and reflect structural characteristics of counties rather than contemporaneous individual attributes, which may introduce temporal mismatch and potential bias in estimated associations. The farmworker variable serves as a coarse proxy for occupational exposure and does not capture variation in employment conditions or vaccination access among individual workers. We note that the 2019 farmworker data do not account for pandemic-related labor disruptions, and future research could leverage pandemic-specific employment data to better assess these effects. Furthermore, the farmworker percentage does not capture other essential workers, such as those in logistics, transportation, or services, who may have faced higher exposure and vaccine requirements in border counties. Despite these limitations, county-level analyses remain useful for identifying geographic and structural patterns that can inform public health planning and resource allocation, especially in border regions where individual-level data are often unavailable.

Given that new COVID-19 variants continue to evolve in the post-pandemic period, threatening public health, effective booster vaccination strategies will require policies that go beyond access alone and explicitly address behavioral fatigue, trust, eligibility timing, and locally specific structural conditions, especially in border and nonmetropolitan settings.

### Limitations

This study has several limitations. As an observational analysis using county-level data, it cannot establish causality or make individual-level inferences. Temporal mismatches exist between datasets, such as COVID-19 outcomes (2022) versus political and socioeconomic data (2010–2019. Although we assume that county-level characteristics such as political orientation, socioeconomic structure, and demographic composition remain relatively stable over time, changes occurring between the earlier measurement period and 2022 may have introduced measurement error. For example, counties experiencing demographic shifts, economic restructuring, migration, or political realignment may have been misclassified relative to their true 2022 characteristics. Such misalignment could attenuate estimated associations, biasing coefficients toward zero or, in some cases, lead to over or underestimation of relationships if structural changes were systematically related to COVID-19 vaccination uptake or mortality.

We used a single 100 km border definition, derived from the La Paz Agreement, which is a policy-based criterion commonly used to designate border counties, without conducting sensitivity analyses with alternative classifications. Racial/ethnic factor was modeled through separate race-specific models rather than a compositional or centered approach, which may limit cross-model comparability. Despite including key variables on politics, socioeconomics, race, and culture, unmeasured factors may influence results. Future research should incorporate individual-level data, enhance temporal consistency, and investigate additional contextual factors to more effectively inform public health and vaccination strategies in border and non-metropolitan areas.

## 6. Conclusions

In conclusion, this study on COVID-19 vaccination rates in four border states reveals that complex patterns of vaccine uptake were associated with various county-level factors. It was found that political affiliation and per capita income consistently showed strong relationships with vaccination rates across dose types, aligning with our previous research [4].

Border counties demonstrated higher vaccination for initial and full doses, while metropolitan counties showed higher uptake primarily for the first dose. The proportion of older adults was only associated with booster uptake while vaccination patterns varied by racial composition across different doses.

These findings highlight the need for targeted strategies in border and non-metropolitan areas, including enhancing access, culturally tailored outreach, and messaging that takes into account political, socioeconomic, and demographic factors. Booster campaigns require distinct approaches from initial rollouts, with a focus on age, risk perception, and local barriers.

## Figures and Tables

**Table 1:** Variables and data sources used in the study of COVID-19 vaccination rate.

Factor	Variable	Description	Data Source
State	State	States (AZ, CA, NM, TX)	
Metro area	Metro status	Metro status = 1, Non-metro status = 0	[44]
Border area	Border county	Border county = 1, Non- Border County = 0	[45]
Employment status	Unemployment	Unemployment rate in 2019	[46]
Political choice	Democrat_pct	Percent of democrat votes, 2020 election result	[47]
	Democrat_1	Democrat = 1, Republican = 0	
Occupation	Farmworker	Percent of workers hired for farm labor	[48]
Area of residence	Rural_pct Urban status	Percent of the county population; Urban if Rural_pct ≤ 50, Rural if Rural_pct > 50 living in rural areas	[49]
Education	HS graduate	Percent of county population who is a high school graduate or higher (5-year estimate) for the population 18 years old and over	[50]
Income	Income	Per capita personal income	[48]
Age	Age_1864	Percent of county resident population aged between 14 and 17	[51]
	Age_65over	Percent of county resident population aged between 18 and 64	
	Age_18over	Percent of county resident population aged 65 years and over	
	Age_median	Percent of county resident population aged 18 years and over	
		Median age of county resident population	
Race/ethnicity	White	Percent of country population by race (White)	[49]
	Black	Percent of country population by race (Black)	
	Asian	Percent of county population by race (Asian)	
	Indian	Percent of county population by race (Indian)	
	Other	Percent of county population by race (other)	
	Hispanic	Percent of county population by Hispanic origin (Hispanic)	
	Non-Hispanic	Percent of county population by non-Hispanic origin (non-Hispanic)	
Vaccination	Administered_Dose1_Pop_Pct (Dose1)	Percent of Total Population with at least one Dose by State of Residence as of 14 September 2022	[44]
	Series_Complete_Pop_Pct (Dose2)	Percent of people who have completed a primary series (have second dose of a two-dose vaccine or one dose of a single-dose vaccine) based on the jurisdiction and county where vaccine recipient lives as of 14 September 2022	
	Booster_Doses_Vax_Pct (Booster)	Percent of people who completed a primary series and have received a booster (or additional) dose as of 14 September 2022	

**Table 2:** Summary statistics (count [%]; mean ± sd) of counties (*N* = 352) and the information by state.

	All	AZ	CA	NM	TX
*N*	352	15	50	33	254
(NA *)	8	0	8	0	0
Metro County	134 [38.1%]	8 [53.3%]	37 [74%]	7 [21.2%]	82 [32.3%]
Border County	44 [12.5%]	4 [26.6%]	2 [4%]	6 [18.2%]	32 [12.6%]
Unemployment (%)	4.07 ± 1.94	6.64 ± 3.28	5.22 ± 3.11	5.44 ± 1.64	3.51 ± 1.09
Democrat_pct (%)	31.57 ± 18.63	43.61 ± 14.34	55.04 ± 15.06	44.79 ± 16.91	24.53 ± 14.15
Farmworker (%)	5.37 ± 5.74	1.57 ± 1.83	1.81 ± 1.99	4.67 ± 5.91	6.39 ± 5.99
Rural_pct (%)	48.98 ± 32.33	34.11 ± 19.75	20.57 ± 20.00	48.42 ± 30.54	55.52 ± 31.90
HS graduate (%)	81.74 ± 8.05	84.48 ± 5.32	83.74 ± 7.19	84.36 ± 5.42	80.85 ± 8.46
Income ($)	49,375 ± 16,205	40,592 ± 5580	57,759 ± 24,555	40,855 ± 9238	49,351 ± 14,391
Age (%)					
14–17 years old	5.36 ± 0.92	5.02 ± 0.88	5.09 ± 0.86	4.93 ± 0.97	5.48 ± 0.90
18–64 years old	57.88 ± 4.14	55.95 ± 5.31	60.32 ± 3.39	56.40 ± 4.28	57.70 ± 4.00
≥65 years old	18.83 ± 5.91	21.70 ± 7.98	17.19 ± 4.73	22.15 ± 7.67	18.55 ± 5.53
≥18 years old	76.71 ± 3.96	77.65 ± 4.03	77.51 ± 4.01	78.55 ± 4.29	76.25 ± 3.82
Median (years old)	39.77 ± 6.25	41.23 ± 8.35	38.72 ± 5.38	42.65 ± 8.06	39.52 ± 5.92
Race/ethnicity (%)					
White	86.57 ± 10.40	78.32 ± 19.40	79.76 ± 11.04	84.94 ± 15.84	88.61 ± 7.49
Black	5.82 ± 5.92	2.36 ± 1.85	4.02 ± 3.23	1.96 ± 1.32	6.88 ± 6.46
Asian	2.45 ± 7.76	1.57 ± 1.15	9.10 ± 9.28	1.24 ± 1.12	1.35 ± 2.08
Indian	2.80 ± 7.32	15.10 ± 20.69	2.53 ± 1.65	9.32 ± 16.14	1.28 ± 0.51
Hispanic	36.31 ± 22.07	31.71 ± 20.83	33.85 ± 18.20	48.69 ± 17.05	35.46 ± 22.97
Vaccination rate (%)					
Dose 1	60.52 ± 16.89	76.31 ± 16.64	74.05 ± 12.98	76.46 ± 15.31	54.85 ± 14.20
Dose 2	52.61 ± 15.38	65.81 ± 16.69	65.87 ± 12.91	64.82 ± 14.01	47.64 ± 12.90
Booster	41.58 ± 8.96	45.33 ± 4.91	53.54 ± 8.42	52.66 ± 7.14	37.56 ± 5.34

* NA indicates the number of counties for which vaccination information is not available.

**Table 3:** Associations between vaccination rate and the counties’ characteristics in the regression analyses (Dose 1).

	(M1)	(M2)	(M3.1)	(M3.2)	(M3.3)	(M3.4)	(M3.5)
	**Model1**	**Model1 + Age_65over**	**Model1+ Age_65over + White**	**Model1 + Age_65over + Black**	**Model1 + Age_65over + Asian**	**Model1 + Age_65over + Indian**	**Model1 + Age_65over + Hispanic**
R-squared	0.792	0.793	0.793	0.806	0.793	0.806	0.793
State (NM *)							
AZ	−1.356	−1.495	−1.788	−1.416	−1.534	−4.029	−1.196
	(0.588)	(0.551)	(0.483)	(0.560)	(0.541)	(0.106)	(0.642)
CA	**−10.388**	**−10.407**	**−10.432**	**−10.150**	**−10.883**	**−8.294**	**−10.084**
	(<0.001)	(<0.001)	(<0.001)	(<0.001)	(<0.001)	(<0.001)	(<0.001)
TX	**−8.465**	**−8.254**	**−8.324**	**−5.286**	**−8.314**	**−6.429**	**−8.033**
	(<0.001)	(<0.001)	(<0.001)	(0.004)	(<0.001)	(<0.001)	(<0.001)
Metro Status	2.035	**2.246**	**2.322**	**2.366**	**2.176**	**3.357**	**2.253**
	(0.054)	(0.040)	(0.035)	(0.026)	(0.047)	(0.002)	(0.039)
Border County	**11.479**	**11.184**	**11.565**	**9.223**	**11.282**	**12.779**	**10.904**
	(<0.001)	(<0.001)	(<0.001)	(<0.001)	(<0.001)	(<0.001)	(<0.001)
Unemployment	0.160	0.154	0.140	0.268	0.180	0.007	0.155
	(0.583)	(0.598)	(0.632)	(0.345)	(0.539)	(0.981)	(0.596)
Democrat_pct	**0.644**	**0.644**	**0.633**	**0.684**	**0.635**	**0.599**	**0.641**
	(<0.001)	(<0.001)	(<0.001)	(<0.001)	(<0.001)	(<0.001)	(<0.001)
Farmworker	0.024	0.004	0.008	−0.060	0.003	−0.010	−0.011
	(0.827)	(0.968)	(0.941)	(0.579)	(0.976)	(0.927)	(0.926)
Rural_pct	−0.017	−0.023	−0.025	−0.015	−0.021	**−0.041**	−0.020
	(0.393)	(0.282)	(0.245)	(0.482)	(0.327)	(0.050)	(0.378)
HS graduate	0.023	−0.003	−0.009	0.031	−0.005	−0.017	0.014
	(0.734)	(0.968)	(0.905)	(0.666)	(0.951)	(0.817)	(0.863)
Income	**9.33 × 10^−5^**	**9.50 × 10^−5^**	**9.55 × 10^−5^**	**7.18 × 10^−5^**	**8.65 × 10^−5^**	**10.48 × 10^−5^**	**9.69 × 10^−5^**
	(0.003)	(0.002)	(0.002)	(0.018)	(0.008)	(0.001)	(0.002)
Age_65over		0.081	0.099	0.024	0.084	0.187	0.093
		(0.431)	(0.354)	(0.808)	(0.415)	(0.068)	(0.378)
Race/ethnicity			−0.035	**−0.386**	0.106	**0.330**	0.016
			(0.502)	(<0.001)	(0.426)	(<0.001)	(0.596)

* refers to reference category. All significant estimates are expressed in bold. Regression coefficient estimates and corresponding *p*-values are reported for each model (*p*-values in parentheses).

**Table 4:** Associations between vaccination rate and the counties’ characteristics in the regression analyses (Dose 2).

	(M1)	(M2)	(M3.1)	(M3.2)	(M3.3)	(M3.4)	(M3.5)
	**Model1**	**Model1 + Age_65over**	**Model1+ Age_65over + White**	**Model1 + Age_65over + Black**	**Model1 + Age_65over + Asian**	**Model1 + Age_65over + Indian**	**Model1 + Age_65over + Hispanic**
R-squared	0.760	0.760	0.761	0.775	0.760	0.779	0.760
State (NM *)							
AZ	0.134	0.046	−0.393	0.123	0.012	−2.749	−0.042
	(0.956)	(0.985)	(0.875)	(0.959)	(0.996)	(0.255)	(0.987)
CA	**−6.195**	**−6.207**	**−6.244**	**−5.956**	**−6.617**	**−3.876**	**−6.302**
	(0.001)	(0.001)	(<0.001)	(0.001)	(<0.001)	(0.037)	(0.002)
TX	**−4.357**	**−4.224**	**−4.330**	**−1.322**	**−4.276**	**−2.211**	**−4.289**
	(0.011)	(0.015)	(0.013)	(0.461)	(0.014)	(0.196)	(0.017)
Metro Status	1.053	1.186	1.300	1.304	1.125	**2.412**	1.184
	(0.308)	(0.267)	(0.226)	(0.208)	(0.294)	(0.022)	(0.268)
Border County	**9.123**	**8.936**	**9.507**	**7.019**	**9.021**	**10.696**	**9.019**
	(<0.001)	(<0.001)	(<0.001)	(<0.001)	(<0.001)	(<0.001)	(<0.001)
Unemployment	0.471	0.467	0.446	**0.579**	0.490	0.305	0.467
	(0.099)	(0.103)	(0.120)	(0.038)	(0.089)	(0.269)	(0.103)
Democrat_pct	**0.611**	**0.611**	**0.594**	**0.649**	**0.603**	**0.561**	**0.612**
	(<0.001)	(<0.001)	(<0.001)	(<0.001)	(<0.001)	(<0.001)	(<0.001)
Farmworker	0.042	0.030	0.036	−0.032	0.029	0.015	0.035
	(0.687)	(0.779)	(0.740)	(0.759)	(0.786)	(0.888)	(0.755)
Rural_pct	−0.004	−0.008	−0.011	0.000	−0.006	−0.028	−0.009
	(0.831)	(0.704)	(0.601)	(0.990)	(0.766)	(0.167)	(0.683)
HS graduate	0.038	0.021	0.012	0.055	0.020	0.006	0.016
	(0.567)	(0.770)	(0.866)	(0.441)	(0.785)	(0.930)	(0.838)
Income	**10.96 × 10^−5^**	**11.07 × 10^−5^**	**11.15 × 10^−5^**	**8.80 × 10^−5^**	**10.34 × 10^−5^**	**12.15 × 10^−5^**	**11.02 × 10^−5^**
	(<0.001)	(<0.001)	(<0.001)	(0.003)	(0.001)	(<0.001)	(<0.001)
Age_65over		0.051	0.077	−0.004	0.054	0.168	0.048
		(0.612)	(0.457)	(0.966)	(0.595)	(0.091)	(0.645)
Race/ethnicity			−0.052	**−0.378**	0.091	**0.364**	−0.005
			(0.304)	(<0.001)	(0.484)	(<0.001)	(0.873)

* refers to the reference category. All significant estimates are expressed in bold. Regression coefficient estimates and corresponding *p*-values are reported for each model (*p*-values in parentheses).

**Table 5:** Associations between vaccination rate and the counties’ characteristics in the regression analyses (Booster).

	(M1)	(M2)	(M3.1)	(M3.2)	(M3.3)	(M3.4)	(M3.5)
	**Model1**	**Model1 + Age_65over**	**Model1 + Age_65over + White**	**Model1 + Age_65over + Black**	**Model1 + Age_65over + Asian**	**Model1** **Age_65over + Indian**	**Model1 + Age_65over** **Hispanic**
R-squared	0.696	0.744	0.749	0.744	0.762	0.745	0.747
State (NM *)							
AZ	**−4.930**	**−5.760**	**−6.362**	**−5.761**	**−5.904**	**−5.939**	**−6.383**
	(0.002)	(<0.001)	(<0.001)	(<0.001)	(<0.001)	(<0.001)	(<0.001)
CA	1.444	1.332	1.282	1.328	−0.390	1.481	0.657
	(0.240)	(0.238)	(0.253)	(0.240)	(0.732)	(0.202)	(0.577)
TX	**−10.910**	**−9.646**	**−9.791**	**−9.692**	**−9.864**	**−9.517**	**−10.106**
	(<0.001)	(<0.001)	(<0.001)	(<0.001)	(<0.001)	(<0.001)	(<0.001)
Metro Status	**−1.992**	−0.732	−0.575	−0.734	−0.985	−0.653	−0.747
	(0.003)	(0.254)	(0.369)	(0.254)	(0.113)	(0.320)	(0.242)
Border County	−0.079	−1.846	−1.063	−1.815	−1.491	−1.733	−1.263
	(0.938)	(0.053)	(0.290)	(0.066)	(0.107)	(0.076)	(0.206)
Unemployment	**−0.373**	**−0.410**	**−0.438**	**−0.411**	−0.314	**−0.420**	**−0.412**
	(0.046)	(0.017)	(0.011)	(0.017)	(0.061)	(0.015)	(0.016)
Democrat_pct	**0.206**	**0.206**	**0.183**	**0.206**	**0.174**	**0.203**	**0.213**
	(<0.001)	(<0.001)	(<0.001)	(<0.001)	(<0.001)	(<0.001)	(<0.001)
Farmworker	**0.168**	0.054	0.062	0.055	0.050	0.053	0.086
	(0.015)	(0.402)	(0.337)	(0.398)	(0.424)	(0.412)	(0.200)
Rural_pct	**0.040**	0.005	0.0005	0.005	0.012	0.003	−0.002
	(0.002)	(0.705)	(0.970)	(0.714)	(0.335)	(0.787)	(0.866)
HS graduate	**0.326**	**0.172**	**0.160**	**0.171**	**0.166**	**0.171**	**0.137**
	(<0.001)	(<0.001)	(<0.001)	(<0.001)	(<0.001)	(<0.001)	(0.004)
Income	0.52 × 10^−5^	1.57 × 10^−5^	1.67 × 10^−5^	1.60 × 10^−5^	−1.53 × 10^−5^	1.63 × 10^−5^	1.18 × 10^−5^
	(0.792)	(0.389)	(0.356)	(0.385)	(0.412)	(0.370)	(0.517)
Age_65over		**0.484**	**0.520**	**0.485**	**0.495**	**0.491**	**0.459**
		(<0.001)	(<0.001)	(<0.001)	(<0.001)	(<0.001)	(<0.001)
Race/ethnicity			**−0.071**	0.006	**0.383**	0.023	−0.034
			(0.019)	(0.904)	(<0.001)	(0.577)	(0.060)

* refers to reference category. All significant estimates are expressed in bold. Regression coefficient estimates and corresponding *p*-values are reported for each model (*p*-values in parentheses).

## Data Availability

The dataset supporting the conclusions of this article is available in the Mendeley Data repository, https://data.mendeley.com/drafts/2wddk5rn9g (accessed on 27 February 2026).

## Appendix A

**Table A1:** Comparison of COVID-19 Vaccination Dose Types.

Characteristic	First Dose (Dose 1)	Second Dose (Dose 2)	Booster Dose
Definition	Initial COVID-19 vaccine	Completion of the primary vaccination series	Additional dose post-primary vaccination
Eligibility	All eligible individuals	Only those who received the first dose	Only those fully vaccinated
Timing	Late 2020 (14 December 2020)	Mid–late 2021	Late 2021 forward (updated as variants emerged)
Population Coverage	Broadest	Subset of first-dose recipients	Subset of fully vaccinated individuals
Sample Selection Implication	Represents the full target population	Filtered by initial compliance	Self-selected, high-engagement population
Analytical Implication	Broad determinants of uptake	Dependent on dose 1 behavior	Selection bias, influenced by the evolving pandemic context

In this study, we define the first dose as the initial COVID-19 vaccination administered to an eligible individual, marking entry into the vaccination program. The second dose refers to the completion of the primary vaccination series, typically for mRNA vaccines (e.g., Pfizer or Moderna). The booster dose, by contrast, is administered after the primary series, intended to maintain immunity over time or address variant-specific challenges.

As shown in Table A1, these three stages of vaccination occurred during distinct phases of the pandemic and involved different policy environments and public attitudes. Notably, booster recipients are a self-selected subset of the vaccinated population, making booster uptake subject to greater sample selection effects and potentially different predictor relationships than those observed for initial doses. Furthermore, the administration of booster doses occurred during a different phase of the pandemic, characterized by evolving public health communication, reduced policy enforcement, and widespread pandemic fatigue, conditions that differentiate booster uptake from initial vaccine doses.
